# Dynamic_Bottleneck Module Fusing Dynamic Convolution and Sparse Spatial Attention for Individual Cow Identification

**DOI:** 10.3390/ani15172519

**Published:** 2025-08-27

**Authors:** Haobo Qi, Tianxiong Song, Yaqin Zhao

**Affiliations:** College of Mechanical and Electronic Engineering, Nanjing Forestry University, Nanjing 210037, China; qihaobo@njfu.edu.cn (H.Q.); songtianxiong@njfu.edu.cn (T.S.)

**Keywords:** Dynamic_Bottleneck, dynamic convolution, sparse-shift attention, individual cow identification

## Abstract

Individual cow identification is a prerequisite for automatically monitoring behavior patterns, health status, and growth data of each cow, and can provide the assistance in selecting excellent cow individuals for breeding. Despite high recognition accuracy, traditional implantable electronic devices such as RFID (i.e., Radio Frequency Identification) can cause some degree of harm or stress reactions to cows. Image-based methods are widely used due to their non-invasive advantages, but these methods have poor adaptability to different environments and target size, and low detection accuracy in complex scenes. To solve these issues, this study designs a Dy_Conv (i.e., dynamic convolution) module and innovatively constructs a Dynamic_Bottleneck module based on the Dy_Conv and S2Attention (Sparse-shift Attention) mechanism. On this basis, we replaces the first and fourth bottleneck layers of Resnet50 with the Dynamic_Bottleneck to achieve accurate extraction of local features and global information of cows. Furthermore, the QAConv (i.e., query adaptive convolution) module is introduced into the front end of the backbone network, and can adjust the parameters and sizes of convolution kernels to adapt to the scale changes in cow targets and input images. At the same time, NAM (i.e., normalization-based attention module) attention is embedded into the backend of the network to achieve the feature fusion in the channels and spatial dimensions, which contributes to better distinguish visually similar individual cows. The experiments are conducted on the public datasets collected from different cowsheds. The experimental results showed that the Rank-1, Rank-5, and mAP metrics reached 96.8%, 98.9%, and 95.3%, respectively. Therefore, the proposed model can effectively capture and integrate multi-scale features of cow body appearance, enhancing the accuracy of individual cow identification in complex scenes.

## Simple Summary

Individual cow identification is a prerequisite for automatically monitoring behavior patterns, health status, and growth data of each cow. Image-based methods have poor adaptability to different environments and target size, and low detection accuracy in complex scenes. To solve these issues, this study designs a Dy_Conv (i.e., dynamic convolution) module and innovatively constructs a Dynamic_Bottleneck module based on the Dy_Conv and S2Attention (Sparse-shift Attention) mechanism. On this basis, we replace the first and fourth bottleneck layers of Resnet50 with the Dynamic_Bottleneck to achieve accurate extraction of local features and global information of cows. Furthermore, the QAConv (i.e., query adaptive convolution) module is introduced into the front end of the backbone network, in order to adapt to the scale changes in cow targets in input images, at the same time, NAM (i.e., normalization-based attention module) attention is embedded into the backend of the network to better distinguish visually similar individual cows. The experiments are conducted on the public datasets collected from different cowsheds. The experimental results showed that the proposed model enhanced the accuracy of individual cow identification in complex scenes.

## 1. Introduction

Individual identification of cows is a key technology for the refined management. It can not only automatically monitor the behavior patterns, health status, and growth data of each cow, but also provide strong technical support for selecting excellent breeding cows. Therefore, in the intelligent management of modern farms and ranches, automatic identification of individual cows is of great significance for ensuring scientific feeding of cows and improving the economic benefits [1,2].

Most cow identification technologies used portable electronic devices such as RFID ear tags to establish cow profiles [3,4]. However, the invasive electronic tags may cause a certain degree of harm and stress response [5,6]. In recent years, computer vision technologies have been widely applied in many fields [7,8,9], including agricultural and livestock management [10,11,12].

The cow identification methods based on machine vision mainly adopted deep learning algorithms to extract the cow’s back pattern features [13,14] or face features [15,16,17], in order to distinguish different individual cows. Some studies were dedicated to scope with the challenges such as lighting changes, various animal postures, partial occlusions and so on. For example, Xiao et al. [18] enhanced the quality of the images under low-light by introducing the image enhancement algorithm MSRCP (i.e., multi-Scale Retinex with chromaticity preservation), and utilized spatial pyramid pooling (SPP) structure to enhance the feature extraction ability of the model. These improvements significantly increased the recognition accuracy under complex lighting conditions. Wang et al. [19] presented a spatial transformation deep feature extraction module integrating the SimAM attention mechanism and ArcFace loss function, in order to improve the model’s adaptability to the challenges such as various animal postures, partial occlusions and complex lighting conditions.

Some studies have utilized multimodal image fusion technologies for individual cow identification [20]. For example, Andrew et al. [21] extracted the local features of cow’s back patterns from RGB-D images for cow identification. Zhao et al. [22] improved the point cloud model PointNet++ to utilize anchor point detection and surface pattern features in depth images for individual identification of Holstein cows. Human re-identification technology has also been used for individual identification of cows [23]. Chen et al. [17] proposed a deep network model GPN that combines three branch modules (middle branch, global branch, and local branch) to extract facial features of cows in different dimensions, and further optimized the extraction of local features through a spatial transformation network (STN). In addition, the spatio-temporal model CNN-BiLSTM has also been applied to individual cattle recognition [24].

However, the above methods are usually designed for specific cowsheds. When applied to different cowsheds with complex environments, these methods may suffer from low recognition accuracy and generalization ability. In addition, with the expansion of breeding scale and the increase in the number of cows, the problem of high similarity in appearance characteristics between individual cows has become more prominent, which poses greater challenges for cow identification [25]. The existing models are difficult to effectively capture enough features to distinguish individual cows, which results in low recognition accuracy.

To address the aforementioned issues, this paper proposes a novel cow identification model based on an innovatively improved Resnet50. Firstly, QAConv was embedded into the front of the Resnet50 structure, in order to adaptively adjust the size and parameters of convolution kernels according to the feature map of the input query image, thereby focusing on the cow’s back features and suppressing irrelevant backgrounds. Then, in the bottleneck structure, we designed a Dy_Conv module and innovatively constructed a Dynamic_Bottleneck module based on the Dy_Conv and S2Attention mechanism. On this basis, we replaced the first and fourth bottleneck layers of Resnet50 with the Dynamic_Bottleneck to achieve accurate extraction of local detail features and global information of cows. Finally, NAM attention mechanism was introduced into the backend of the Resnet50 structure to achieve feature fusion in the channels and spatial dimensions, which was beneficial for better distinguishing visually similar individual cows.

The primary contributions of the study can be summarized as follows:

- In order to effectively extract the multi-scale features, we designed a dynamic convolution module Dy_Conv with a parallel structure of multiple branches. Dy_Conv generates feature maps with receptive fields of different sizes, allowing the model to simultaneously focus on the global features and back pattern features of cows.
- We constructed the Dynamic_Bottleneck module combining Dy_Conv with S2Attention, and replaced the 1st and 4th bottleneck layer of Resnet50, reducing computational complexity while efficiently fusing the multi-scale features. This helped the model to be applied to different cowsheds with complex environments.
- We embedded QAConv into the front of Resnet50, which adjusted the parameters and sizes of convolution kernels to adapt to the scale changes in cow targets in input images.
- To enhance the perception of local details, NAM attention mechanism was introduced into the backend of Resnet50 to achieve the feature fusion in the channels and spatial dimensions, which contributed to better distinguish visually similar individual cows.

## 2. Materials and Methods

### 2.1. Experimental Dataset

**Figure 1:**
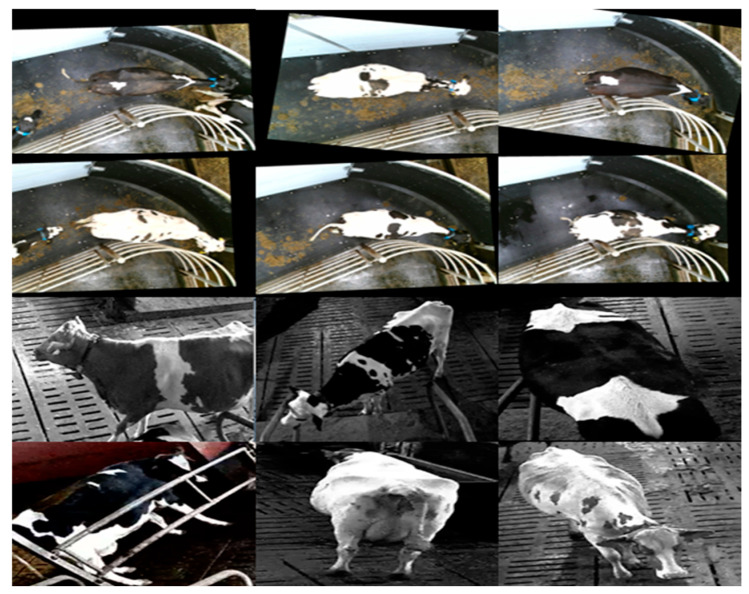
The example images of the dataset. The samples in the top two rows are taken from the milking room; the bottom rows are taken from the cowshed.

The images in the experimental dataset are derived from two public datasets. One is provided by the University of Bristol, containing 940 images from 89 cow individuals in the milking room (https://data.bris.ac.uk/data/dataset/2yizcfbkuv4352pzc32n54371r (accessed on 6 December 2024)). The other dataset consists of cow surveillance videos from different cowsheds (available at https://doi.org/10.5281/zenodo.3981400 (accessed on 20 June 2024)). In the dataset, the images in a video recorded by a fixed camera have the same background, and each video has a short duration, so most individual cows are only captured at a fixed angle. To validate the model more convincingly, we manually cropped 206 images of 30 cow individuals taken from various angles. The example images are displayed in Figure 1.

Referring to the dataset partitioning method of the pedestrian re-identification, we divided the dataset into a training set and a testing set. The testing set is further divided into the Gallery set and the Query set, where the Gallery set refers to the cows with a known identity number, and the Query set contains the cows to be identified by the model. The ratio of training set, Gallery set, and Query set is 6:3:1.

### 2.2. Methods

**Figure 2:**
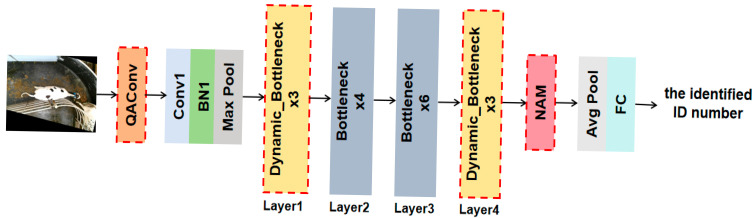
Overall framework of the cow individual recognition model. The QAConv module is introduced to adapt to the scale changes in the target. The proposed Dynamic_Bottleneck replaces the first and fourth bottleneck layers for the accurate extraction of local features and global information of cows. NAM is embedded for the feature fusion in the channel-spatial dimension.

We designed the dynamic convolution module Dy_Conv and innovatively constructed the dynamic bottleneck block Dynamic_Bottleneck based on Dy_Conv and S2Attention mechanism. On this basis, we replaced the first and fourth bottleneck layers of ResNet50 with the Dynamic_Bottleneck. The overall framework of the cow individual recognition model is shown in Figure 2. Firstly, the QAConv module was introduced at the front end of the network structure to adapt to the scale changes in the targets by adaptively adjusting the parameters and size of convolution kernels based on the input cow images. Next, a four-layer bottleneck structure was used to gradually extract high-level semantic features of cows. In the Dynamic_Bottleneck structure, the Dy_Conv consists of a depthwise convolution and two dilated convolutions with different dilation rates. The parallel branch structure can achieve the accurate extraction of local features and global information of cows. The S2Attention mechanism adopts the feature channel compression and the region sparsity sampling strategy to achieve multi-scale feature interaction and fusion. Then, by introducing the NAM attention mechanism in the backend of the network, the local features representation of the cow’s back pattern were strengthened in the spatial dimension, thus the feature fusion in the channel-spatial dimension can better distinguish visually similar individual cows. Finally, the proposed model compressed the spatial dimension through a global average pooling layer, and obtained feature vectors with global representation ability, which were then mapped to the classification space through a fully connected layer.

#### 2.2.1. Low-Level Feature Extraction Based on QAConv

**Figure 3:**
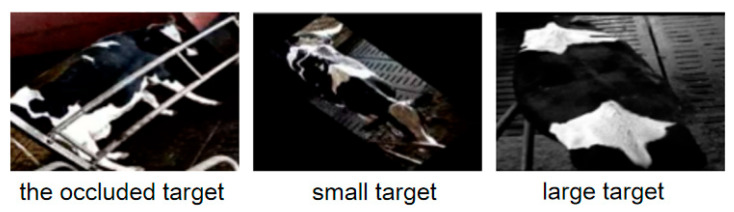
The challenging cow targets.

The convolution kernel with the fixed parameters and size has the limitations when facing the challenging images such as occlusions or targets of different sizes, as shown in Figure 3, thus the fixed receptive fields are difficult to adapt to scale changes in targets. The size and parameters of QAConv’s convolution kernel can be adaptively adjusted according to the feature maps of the input query image, enhancing the model’s attention to the cow’s back characteristics while suppressing the interference of irrelevant backgrounds.

**Figure 4:**
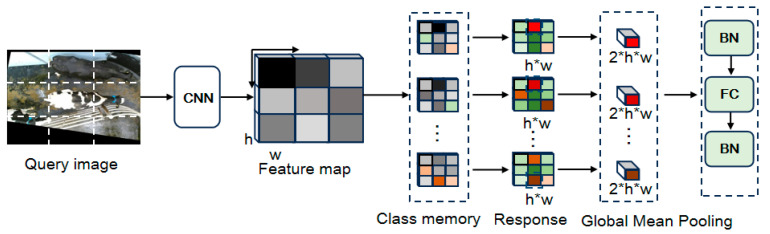
The structure of QAConv.

The structure of QAConv [26] is shown in Figure 4. The query image of individual cows is input into the network, and CNN backbone networks are used to extract feature maps of the query image and candidate Gallery images. Class Memory extracts a fixed size local region at each spatial position of the feature map to adaptively adjust the convolution kernel parameters. In the Response phase, the local matching scores are calculated by the pointwise convolution of the adaptive convolution kernel and feature map. Next, Global Mean Pooling aggregates local matching scores into global matching scores while reducing dimensionality. Finally, BN-FC-BN converts the matching score into the similarity measure to match the query image and candidate images.

#### 2.2.2. Dynamic_Bottleneck for Multi-Scale Feature Extraction

The Bottleneck of the original ResNet50 has the limited ability to capture global features due to the fixed receptive fields. In addition, the feature fusion relies solely on simple residual connections, which lacks the ability to dynamically adjust the weights of feature channels. Compared with the standard convolution, dynamic convolution can adjust convolution kernel parameters based on input feature maps, thereby enhancing the feature expression ability of the model and making it more suitable for complex tasks.

In the network structure of individual cow recognition, we constructed the Dynamic_Bottleneck and replaced the first and fourth bottleneck layers of ResNet50 with Dynamic_Bottleneck, as shown in Figure 1. Compared with the original bottleneck, the Dynamic_Bottleneck in the Layer1 can preserve more low-level visual features and avoid local detail loss, and the Dynamic_Bottleneck of Layer4 can expand the receptive fields through the Dy_Conv with a multi-branch structure, enhancing the model’s ability to integrate global information. The number of channels in Layer2 and Layer3 is relatively small, and the performance improvement brought by Dynamic_Bottleneck is limited. Moreover, the flexibility of dynamic convolution is not conducive to stable modeling of context, so the standard bottleneck structure is retained in Layer2 and Layer3.

**Figure 5:**
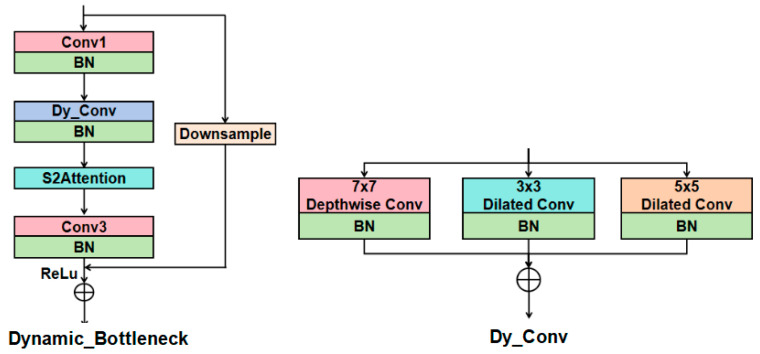
The structure of Dynamic_Bottleneck. Dy_Conv and S2Attention are embedded into the Bottleneck. Dy_Conv is a parallel structure consisting of a depthwise convolution branch and two dilated convolution branches.

The structure of the proposed Dynamic_Bottleneck is shown in Figure 5. Dynamic_Bottleneck was constructed by introducing Dy_Conv and S2Attention into the bottleneck of ResNet50. Dy_Conv is a parallel structure consisting of a depthwise convolution branch and two dilated convolution branches. Depthwise convolution is used to capture local features, 5 × 5 dilated convolution expands the receptive fields to capture large-scale contextual information, and 3 × 3 dilated convolution is used to enhance local details. Therefore, the Dy_Conv can generate feature maps with receptive fields of different sizes. Finally, the weighted fusion features include both local features and contextual information, allowing the model to simultaneously focus on the global features and back pattern features of cows, which is very beneficial for individual cow identification.

**Figure 6:**
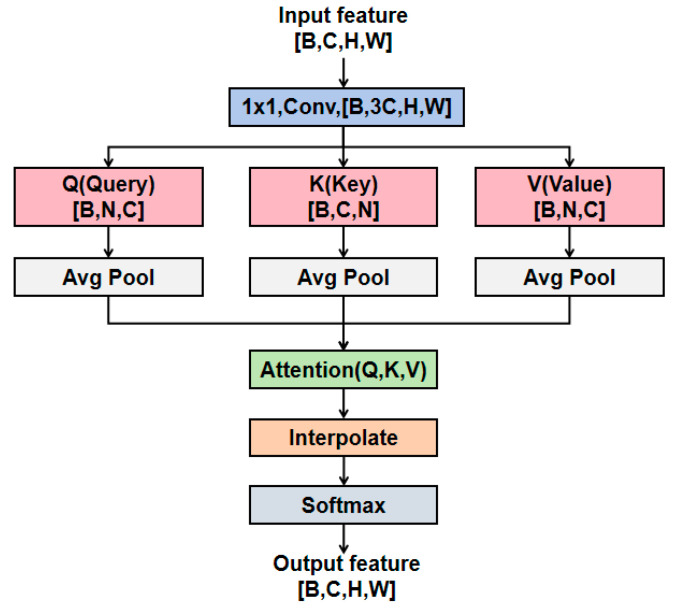
The structure of S2Attention. B is the batch size, C represents the number of channels, H and W, respectively, represent the height and width of the feature maps, $N=\;\frac{H}{stride}\times \frac{W}{stride}\;$, where stride is set as 2 for the balance of feature representation and computational cost.

The S2Attention module [27] achieves the attention modeling of key regions using the sparse sampling strategy. In addition, the multi-scale feature interaction based on the down-sampling and up-sampling operations enhances the model’s perception ability of cow targets of different sizes. As shown in Figure 6, S2Attention first uses 1 × 1 convolution to expand the channels of the feature map by three times, and splits it into Query, Key, and Value vectors followed by average pooling, thereby constructing multi-scale feature representation. Then, the attention is calculated in the down-sampled feature space. Finally, the initial spatial dimensions are restored by the up-sampling operation.

#### 2.2.3. NAM for the Fusion of Channels and Spatial Features

The NAM attention mechanism [28] computes channel attention and spatial attention in parallel. The channel attention sub-module dynamically adjusts the weight of each channel to pay more attention to important channels. The spatial attention sub-module generates pixel attention weights to focus on the pixels with the most relevant information in the cow image. This fine-grained spatial attention mechanism enhances the local feature representation of the cow’s back patterns. Therefore, the NAM attention mechanism integrates features in the channels and space dimensions, which is beneficial for better distinguishing visually similar individual cows and improving the accuracy of individual cow recognition.

**Figure 7:**
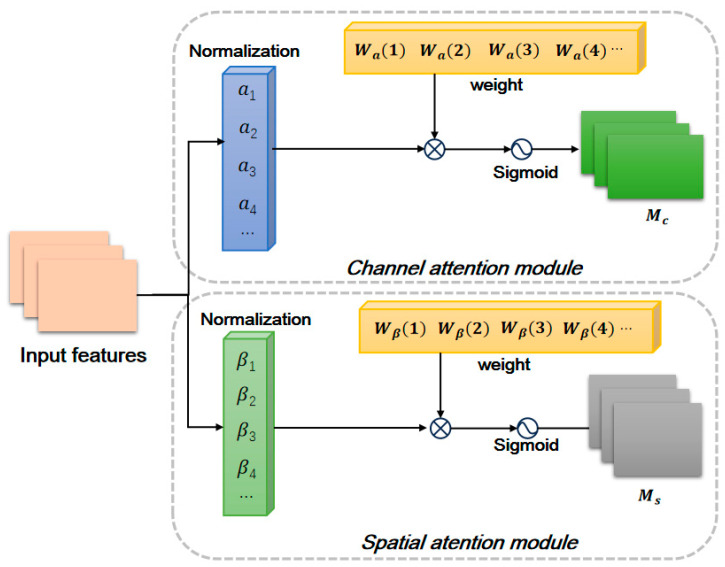
The structure of NAM.

The NAM attention mechanism [28] calculates the Channel attention module and Spatial attention module in parallel. As shown in Figure 7, the NAM attention mechanism normalizes the feature map. The normalization formula of the channel attention module is as follows:

(1) $$B_{out}=BN(B_{in})=a\frac{B_{in}-\mu_{B}}{\sqrt{\sigma_{B}^{2}+\epsilon }}+\beta$$

where the $B_{in}$ and $B_{out}$ denote the input feature map and output feature map, respectively. $\mu_{B}$ and $\sigma_{B}$ are batch data of mean and variance, respectively. a and β are the trainable affine transformation parameters (i.e., scale factor and shift factor) for each channel, respectively [28,29]. Then, the results are normalized to reduce the numerical differences between different channels. The normalized channel weights is shown in Equation (2):

(2) $$W_{a}(i)=\frac{\alpha_{i}}{\sum_{j=0}\alpha_{j}}$$

where $\alpha_{i}$ is the scale factor of each channel and $W_{a}$ is the weight. The normalized result is calculated by using the Sigmoid function, in order to calculate the output of the NAM channel attention module $M_{c}$. The calculation formula can be computed by Equation (3):

(3) $$M_{c}=Sigmoid\left(W_{\alpha }\left(BN\left(B_{in}\right)\right)\right)$$

Similarly, pixel normalization can be performed on the spatial attention module to obtain the pixel normalized weights, and then the Sigmoid function can be used to calculate the output $M_{S}$ of the channel attention module of NAM.

## 3. Results

### 3.1. Experimental Details

The experiments are implemented on the NVIDIA RTX 3060 GPU and the PyTorch 1.71 framework. The input image size was resized as 384 × 192. Several data enhancement methods were applied to augment the number of training samples, such as random cropping, masking, horizontal flipping, and mirroring. In order to compare all the models fairly, we used the same hyper-parameters for training them, namely, 10 image padding, 16 batch sizes, and 0.9 momentum. The training period was 80 epochs, with an initial learning rate of 0.0001. During training, the network utilized the Adam optimizer and triplet loss function. Cumulative Matching Characteristics (CMC) and Mean Average Precision metrics were used for evaluating the experimental results, where we used Rank-1 and Rank-5 to evaluate the CMC.

### 3.2. Ablation Experiments

To validate the effectiveness of the improvements on individual cow recognition, we conducted the ablation experiments on QAConv, NAM attention, Dy_Conv, and S2Attention. As seen in Table 1, the evaluation metrics Rank-1 and mAP of the baseline model ResNet50 are only 89.7% and 90.8%, respectively. After QAConv was introduced, the size and parameters of the convolution kernels could be adaptively adjusted, enhancing the model’s adaptability to target scale changes in different input images, which resulted in a 4.9% and 2.9% improvement in Rank-1 accuracy and mAP metrics, respectively. However, when the bottleneck blocks of Layer1 and Layer4 were replaced with Dynamic_Bottleneck, the indicator Rank-1 actually decreased by 0.3%, although both Rank-5 and mAP values had slight improvements. The decrease in Rank-1 means that the model reduced the performance in distinguishing individual cows with similar appearance features, because Dy_Conv did not integrate the features well after extracting features from different receptive fields. Therefore, the model’s performance is further reduced when all the four Dynamic_Bottleneck layers were used to replace the original bottleneck layers. After incorporating S2Attention into Dynamic_Bottleneck to effectively fuse these features, all three evaluation metrics improved, especially the value of Rank-1 increased by 1.4%. Furthermore, compared with 2 Dynamic_Bottleneck layers with S2Attention, 4 Dynamic_Bottleneck layers with S2Attention had lower performance despite less parameters. The NAM attention mechanism enhanced the local feature representation of cow back patterns through spatial attention; therefore, it improved the Rank-1, Rank-5, and mAP values by 1.1%, 1.3%, and 0.6%, respectively. To reduce the number of parameters, we down-sampled the Layer2 and Layer3 bottlenecks of ResNet50.

**Figure 8:**
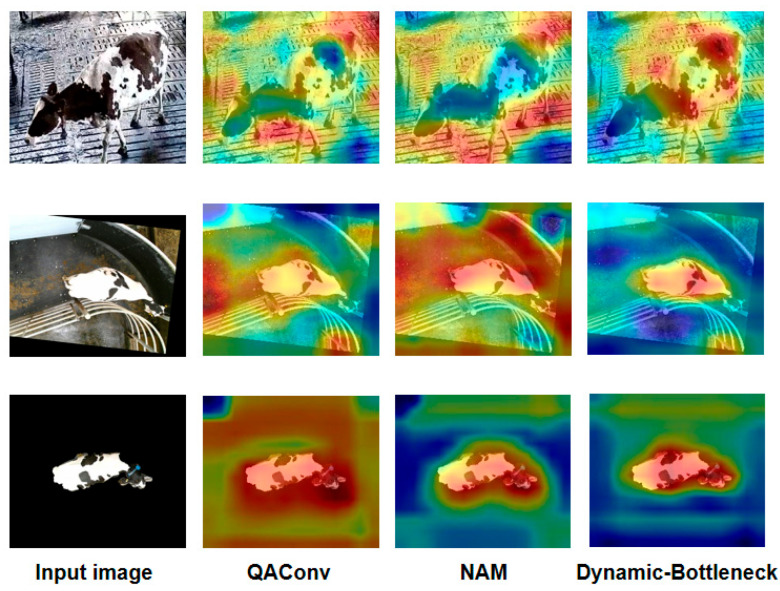
Visualization of the feature heatmaps.

Figure 8 visualizes the feature heatmaps of three modules (QAConv, Dynamic_Bottleneck, and NAM) introduced into ResNet50. As seen in Figure 8, the QAConv module focuses more on the cow’s back pattern features, but it also pays attention to many irrelevant backgrounds. The embedding of NAM reduces background attention to a certain extent, especially in the third row of Figure 8, but sometimes it can also cause the model to not pay enough attention to the key features of cows, as shown in the first row of Figure 8. Furthermore, after replacing the bottleneck of Resnet50 with Dynamic_Bottleneck, the Dy_Conv in Dynamic_Bottleneck extracts multi-scale features based on different receptive fields, and the S2Attention module achieves multi-scale feature interaction during the feature fusion. Therefore, the output feature heatmap pays high attention to the back pattern of cows, while significantly reducing the attention to irrelevant backgrounds.

### 3.3. Model Evaluation

To validate the performance of the proposed cow individual recognition model, we compared it with the existing mainstream animal re-identification models (TigerReID, Part-Pose ReID) and pedestrian re-identification models (BDB and OSNet-AIN). All the experiments were conducted on the two datasets described in Section 2.1, and the datasets were divided according to the same standards, in order to compare all the models fairly. As shown in Table 2, and the proposed method outperforms the comparison methods in all the evaluation metrics. Compared with the optimal method OSNet-AIN, Rank-1, Rank-5, and mAP values of the proposed method are 3.1%, 1.1%, and 1.4% higher, respectively. The experimental data sources from different cowsheds, especially from actual cowshed scenes where the appearance of individual cows is very similar, and the shooting light is relatively dark, which also verifies the generalization ability of our method in different application scenarios.

**Table 2:** Comparison with advanced identification methods.

Method	Rank-1	Rank-5	mAP
TigerReID [30]	86.1%	93.3%	87.5%
Part-Pose ReID [31]	89.7%	94.7%	91.7%
BDB [32]	89.7%	95.7%	89.7%
OSNet-AIN [33]	93.7%	97.8%	93.9%
Ours	96.8%	98.9%	95.3%

### 3.4. Visualization of Feature Maps

**Figure 9:**
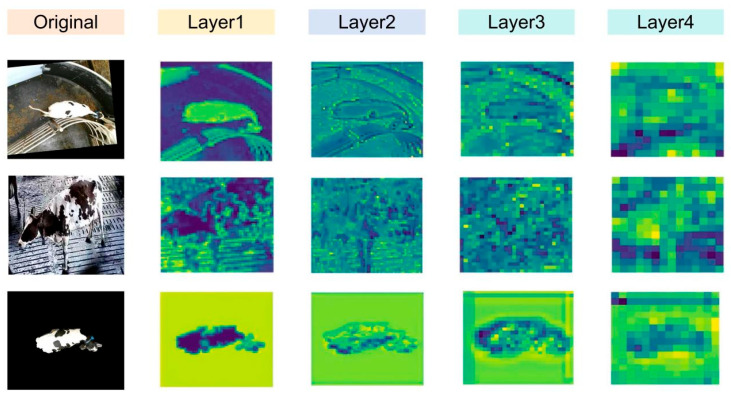
Visualization of the feature map of bottleneck structure. The feature maps are output by the last convolution in each layer. Layer1 and Layer4 used the Dynamic_Bottleneck proposed in this study, while Layer2 and Layer3 used the bottleneck of Resnet50.

We visualized the feature map of each bottleneck layer of the proposed model (shown in Figure 2). Layer1 and Layer4 used the Dynamic_Bottleneck proposed in this study, while Layer2 and Layer3 used the bottleneck of Resnet50. The feature map output by the last convolution in each layer is shown in Figure 9. Layer1 and Layer2 extracted low-level features such as cow contour features and fine-grained features like back pattern texture; Layer3 and Layer4 extracted high-level semantic information such as the topological structure and spatial distribution of cow back patterns, in order to distinguish the feature differences in different cow individuals. This process achieved a hierarchical mapping from low-level visual patterns to high-level semantic abstractions.

## 4. Discussion

### 4.1. Advantage of Dynamic_Bottleneck Design

Dynamic_Bottleneck combines the dynamic convolution module Dy_Conv and S2Attention. As shown in Table 1, after Dynamic_Bottleneck is used in the bottleneck structure, the model’s metrics Rank-1, Rank-5, and mAP are significantly improved. From the visualization feature maps in Figure 8 and Figure 9, it can be seen that the model simultaneously focuses on the global features and back pattern features of cows. This indicates that the Dynamic_Bottleneck module effectively extracts and integrates local detail features and global contextual information, providing rich feature representations for individual cow recognition.

Compared with the original bottleneck of ResNet50, Dynamic_Bottleneck requires a lower number of parameters in practical applications. This is because the Dy_Conv block in Dynamic_Bottleneck uses depthwise separable convolution and parallel branch design, which has much fewer parameters than traditional convolution, and the three channels are independently processed by three parallel branches. Therefore, compared to 3 × 3 convolution, Dy_Conv requires fewer parameters. Especially for the Layer4 Dynamic_Bottleneck with a large number of channels, the parameters has a significant decrease.

To verify the feature interaction and fusion ability of S2Attention, we replaced S2Attention with SE (Squeeze and Excitation) attention mechanism. The experimental results are shown in the third and fourth rows of Table 3. Although the parameters of S2Attention module in Dynamic_Bottleneck slightly increased compared to SE attention module, the values of Rank-1, Rank-5, and mAP increased by 1.1%, 2.1%, and 1.2%, respectively. This is because the S2Attention module maintains attention modeling of key regions through sparse sampling strategy. In addition, multi-scale feature interaction is achieved through down-sampling and up-sampling operations, enhancing the model’s perception ability of multi-scale features.

### 4.2. Comparison Between NAM and Mainstream Attentions

In addition, we also compared the effects of NAM attention and two other mainstream attention mechanisms on individual cow recognition. To ensure the fairness of the experiment, all tests were conducted using the same hyper-parameter settings. As shown in Table 3, compared with the other attention mechanisms, NAM performs excellently in improving model performance. NAM attention effectively integrates feature information in the channel-space dimension through parallel computation of channel attention and spatial attention, thereby better distinguishing visually similar individual cows. In addition, NAM also has a relatively lower number of parameters.

**Figure 10:**
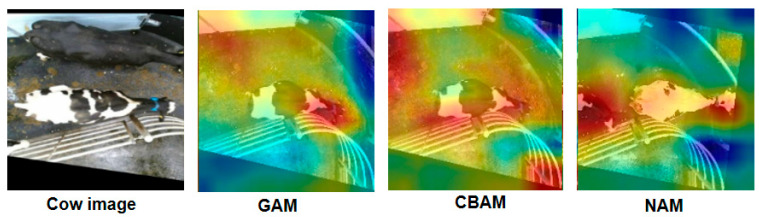
The heatmaps of different attention modules.

Figure 10 visualizes the heatmaps of different attention modules. GAM and CBAM simultaneously focus on the body of cows and a large number of background areas, which is not conducive to capturing key features of cows and thus reduces the accuracy of identifying individual cows. Compared with GAM and CBAM, NAM focuses more on the torso area of cows, and reduces interference with irrelevant backgrounds.

### 4.3. Application on Cowsheds

**Figure 11:**
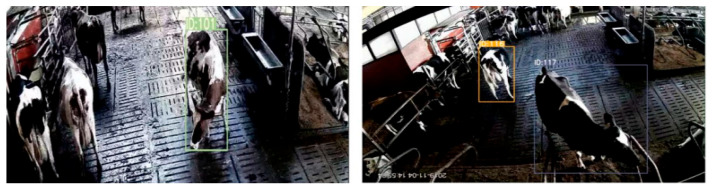
Sample images of identifying multiple individual cows in a video frame. The cows are located by YOLOv8, and their ID numbers are identified by the proposed model.

For the dataset in the milking room, we directly adopt the proposed method for individual cow recognition. In another dataset, each cow video frame contains multiple cows. We labeled a small portion of images in the dataset and fine-tuned the pretrained YOLOv8 to detect the cow targets. Next, we cropped the cow targets in the bounding boxes generated by YOLOv8. The cropped images were regarded as the images in Query set, then, the cow individual recognition model matched the cropped cow target images with the images in Gallery set, in order to identify the ID number of the cow in the video frame. Since we selected 30 individual cows, only these 30 cows in the dataset can be assigned ID numbers, as shown in Figure 11.

### 4.4. Accuracy and Limitations

**Figure 12:**
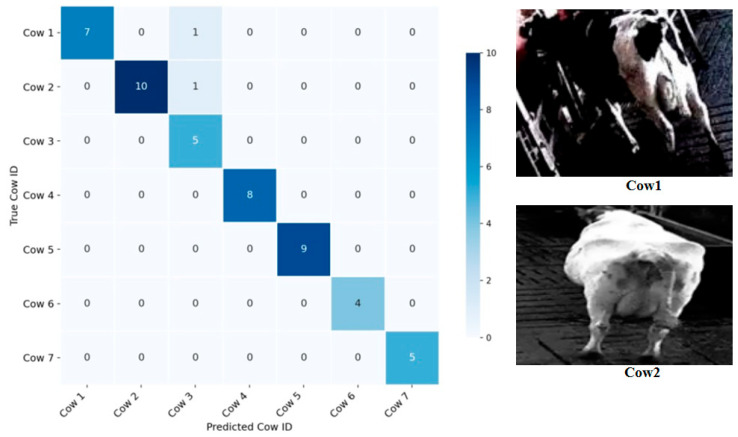
Confusion matrix for individual identification of cows. (**Left**): confusion matrix; (**Right**): two mis-identified cow images.

Accuracy is also used to evaluate the performance of the proposed model. The confusion matrix is shown in Figure 12. We randomly selected the IDs of seven cows, totaling 50 cow images. The number of images for each individual cow is shown in Table 4. As seen from the left image of Figure 12, 48 out of 50 cow images were correctly identified, thus the identification accuracy was 96%. Among them, each of ID Number Cow1 and Cow2 had one image given the wrong ID (namely Cow3). The two misidentified cow images are shown on the right side of Figure 10. The black areas of the Cow1 body are difficult to be distinguished from the backgrounds due to the shooting angle and dim lighting. The body of the Cow2 is perpendicular to the camera lens plane, which makes it impossible to extract its back pattern features.

## 5. Conclusions

This article presented a novel individual cow recognition method to adapt to complex scenarios in different cowsheds, as well as to distinguish visually similar individual cows. The QAConv module was introduced to front-end of the backbone network to address the scale changes in cow targets in the input image. The Dynamic_Bottleneck module based on Dy_Conv and S2Attention mechanism achieved accurate extraction of local detail features and global information of cows. NAM attention was embedded into the backend of the network for the feature fusion in both channel and spatial dimensions. The experiment verified that the proposed method had better performance and fewer parameters than the state-of-the-art re-identification methods. The proposed method can reduce the interference of complex backgrounds and improve the recognition accuracy of visually similar individual cows. In addition, this method can be extended to other livestock breeding scenarios.

However, the method proposed in this article has some limitations. Due to the shooting angle, the black patches on the cow body almost blend in with the dark background in low light environments, as a result, these local features are not accurately extracted by the model. In addition, when the body of a cow is at a vertical angle to the camera lens, most of the patterns on its torso are occluded except for the face or tail. Cows captured at this angle are often misidentified.

In future research, we will combine the advanced image restoration techniques into the individual cow recognition method, in order to solve the problem of individual cow identification in low light images. We will also use multiple cameras to capture images of the same cow from different angles for model training, and explore fusion strategies for multi angle input images to improve the accuracy of individual cow recognition.

## Figures and Tables

**Table 1:** Ablation experiment.

Model	Rank-1	Rank-5	mAP	Parameters
ResNet50	89.7%	95.6%	90.8%	23,508,032
ResNet50 + QAConv	94.6%	96.8%	93.7%	23,508,317
ResNet50 + QAConv + Dynamic_Bottleneck × 2 (no S2Attention)	94.3%	97.2%	94.1%	16,473,629
ResNet50 + QAConv + Dynamic_Bottleneck × 4 (no S2Attention)	93.1%	95.5%	92.8%	12,526,848
ResNet50 + QAConv + Dynamic_Bottleneck × 2 (with S2Attention)	95.7%	97.6%	94.7%	19,675,421
ResNet50 + QAConv + Dynamic_Bottleneck × 4 (with S2Attention)	94.9%	97.4%	93.6%	17,571,840
ResNet50 + QAConv + Dynamic_Bottleneck + NAM (with 2 Dynamic_Bottleneck layers)	96.8%	98.9%	95.3%	19,679,517

**Table 3:** Comparison of Attention Mechanisms.

Dynamic_Bottleneck	Attention Mechanism	Rank-1	Rank-5	mAP	Parements
(with S2Attention)	CBAM	92.7%	96.4%	93.4%	23,512,128
	GAM	93.3%	97.8%	93.7%	23,843,705
	NAM	96.8%	98.9%	95.3%	19,679,517
(with SE Attention)	NAM	95.7%	96.8%	94.1%	16,669,248

**Table 4:** The number of images for each individual cow.

ID Number	Cow1	Cow2	Cow3	Cow4	Cow5	Cow6	Cow7	Total
**Number of images**	8	11	5	8	9	4	5	50

## Data Availability

The data presented in this study are available on request from the corresponding author.
